# Risk factors for retinopathy in hemodialysis patients with type 2 diabetes mellitus

**DOI:** 10.1038/s41598-020-70998-9

**Published:** 2020-08-25

**Authors:** Michael Müller, Carl-Ludwig Schönfeld, Tanja Grammer, Vera Krane, Christiane Drechsler, Bernd Genser, Thomas Kohnen, Christoph Wanner, Winfried März

**Affiliations:** 1 Department of Ophthalmology, Goethe-University, Theodor Stern Kai 7, 60590 Frankfurt am Main, Germany; 2 Herzog Carl Theodor Eye Clinic, Munich, Germany; 3 Mannheim Institute of Public Health, Social and Preventive Medicine, Medical Faculty Mannheim, University of Heidelberg, Heidelberg, Germany; 4 Division of Nephrology, Department of Medicine, University Clinic Würzburg, Würzburg, Germany; 5 Clinical Institute of Medical and Chemical Laboratory Diagnostics, Medical University of Graz, Graz, Austria; 6 Synlab Academy, Synlab Services GmbH, Mannheim, Germany

**Keywords:** Type 2 diabetes, Retinal diseases, Endocrinology, Kidney diseases

## Abstract

There is limited knowledge on the prevalence and risk factors of diabetic retinopathy (DR) in dialysis patients. We have investigated the association between diabetes mellitus and lipid-related biomarkers and retinopathy in hemodialysis patients. We reviewed 1,255 hemodialysis patients with type 2 diabetes mellitus (T2DM) who participated in the German Diabetes and Dialysis Study (4D Study). Associations between categorical clinical, biochemical variables and diabetic retinopathy were examined by logistic regression. On average, patients were 66 ± 8 years of age, 54% were male and the HbA1c was 6.7% ± 1.3%. DR, found in 71% of the patients, was significantly and positively associated with fasting glucose, HbA1c, time on dialysis, age, systolic blood pressure, body mass index and the prevalence of other microvascular diseases (e.g. neuropathy). Unexpectedly, DR was associated with high HDL cholesterol and high apolipoproteins AI and AII. Patients with coronary artery disease were less likely to have DR. DR was not associated with gender, smoking, diastolic blood pressure, VLDL cholesterol, triglycerides, and LDL cholesterol. In summary, the prevalence of DR in patients with type 2 diabetes mellitus requiring hemodialysis is higher than in patients suffering from T2DM, who do not receive hemodialysis. DR was positively related to systolic blood pressure (BP), glucometabolic control, and, paradoxically, HDL cholesterol. This data suggests that glucose and blood pressure control may delay the development of DR in patients with diabetes mellitus on dialysis.

## Introduction

Diabetes mellitus (DM) is more common in Western countries. The chronic course of DM and multiple end-organ damages like diabetic nephropathy, neuropathy, and retinopathy (DR) compromises the quality of life of affected patients and produces increased health care costs^1^.

Type 2 diabetes mellitus (T2DM) is the most common single disease causing end-stage renal disease (ESRD) which results in hemodialysis. Diabetic nephropathy has been reported in approximately 40% of patients who need renal replacement therapy^2^. Patients with T2DM on hemodialysis show a higher prevalence of co-morbidities and poorer outcome in comparison to non-diabetic patients on dialysis^3^. This is reflected by a five-year survival rate of only 35%^4^.

DR is a common result of DM. The prevalence rate of DR in T2DM-patients not receiving dialysis varies within a broad range: it is reported between 34.6^5^ and 64%^6^. DR is one of the main reasons for blindness in the Western hemisphere. Global causes of visual impairment and blindness are 1% due to diabetic retinopathy^7^. The annual incidence rate of blindness due to DR is between 1.2 and 2.1 per 100.000^8^.

The life expectancy of T2DM patients on dialysis has recently improved due to technical enhancements, e.g. refined control of the dialysis-machines, high-flux and bio-compatible membranes, more flexible catheters and pre-mounted stents with lower profile^9,10^. Therefore, quality of life in these patients becomes more critical. Although DR is not life-threatening, the quality of life is obviously superior without visual problems. We, therefore, became interested in the metabolic determinants of DR in hemodialysis patients. While there are studies of the coincidence of DR and nephropathy, e.g. Banerjee et al.^11^, lesser amount of information is available with regard to the prevalence of DR in T2DM dialysis patients^12,13^.

We investigated the prevalence and risk factors of diabetic retinopathy in patients on hemodialysis due to diabetes mellitus-related ESRD using data from the German Diabetes Dialysis Study (4D Study: Die Deutsche Diabetes Dialyse Studie), which evaluated atorvastatin 20 mg daily compared to placebo in 1,255 patients with T2DM on maintenance hemodialysis^14^. We wondered, if there would be patients with ESRD needing hemodialysis, but not having DR, because the DM related vessel damage is not only likely to be found in the kidney, but also effects retinal vessels^11^. For the patients without DR, we were interested what distinguished them from other patients with DR.

## Materials and methods

### Study design and participants

We investigated the cross-sectional data collected at the time of enrollment of the participant study and before starting study medication. Thus, we were not able to make any predication on the role of treatment alterations, e.g. atorvastatin, on the occurrence of DR.

The 4D study has previously been described in detail^15^. In summary, it was a prospective randomized controlled trial including 1,255 patients with T2DM, age 18–80 years, and on hemodialysis for less than 2 years. The study was designed to examine the effects of atorvastatin compared to placebo on adverse cardiovascular events.

Patients were recruited between March 1998 and October 2002 from 178 dialysis centers in Germany. The primary endpoint of the 4D study was defined as a composite of death from cardiac causes, fatal or nonfatal stroke, and nonfatal myocardial infarction (MI), whichever occurred first (composite cardiovascular endpoint; CVE).

The protocol was approved by the ethics committee of the Medical Faculty at the Medical University of Würzburg, Germany (Address: Ethik-Kommission der Universität Würzburg, Institut für Pharmakologie und Toxikologie, Versbacher Str. 9, 97078 Würzburg, Germany) at the coordinating center (Medical University of Würzburg, Germany) and 29 regional ethics committees responsible for the study sites. Prior to the study, all patients provided their written informed consent. The current evaluation is completely covered by the initial and written informed consent of the study participants. Upon granting the study, the ethics committee of the University of Würzburg has been presented the record details relating to the study, which included information on retinopathy. The item “retinopathy” was also included in the baseline paper (page 262, Results, *Medical and Drug History*^15^). The current paper does not go beyond the original evaluation framework that was approved by the University of Würzburg.

### Data collection

Details regarding age, gender and smoking status was obtained through patient interviews. Smoking status was classified as never, former, or current.

Using a standardized questionnaire, the patients´ nephrologists or ophthalmologist reported the presence of DR. The diagnosis of DR was based on clinical examination. A differentiation of DR stages (e.g. non-proliferative or proliferative) by additional ophthalmological examination was not completed. Furthermore, blindness is defined as a visual acuity lower than 3/60 (World Health Organization’s definition of blindness^7^: 3/60 = 0.05 decimal scale; normal vision is 20/20 = 1.0), was reported by the patients’ ophthalmologist. Uniformity between questionnaire and the source data in the patient’s records was validated using approved study monitors.

Other types of microvascular disease were recorded, such as peripheral vascular disease (PVD), nephrotic syndrome (NS), polyneuropathy (PN), diabetic gangrene (DG), and macrovasculopathies similar to myocardial infarction (MI), stroke/TIA, percutaneous transluminal coronary angioplasty (PTCA), definite coronary artery disease (CAD), coronary artery bypass graft (CABG).

Blood pressure was measured in a sitting position. The measurements of glucose, HbA1c, total cholesterol, high-density lipoprotein (HDL) cholesterol, low-density lipoprotein (LDL) cholesterol, and triglycerides have been described^14,15^.

Body mass index (BMI) uses a weight-to-height ratio (BMI = kg/m^2^). All laboratory measurements of the 4D study were performed locally at the Department of Clinical Chemistry, University of Freiburg, Germany. Blood samples were taken prior to the start of dialysis sessions and administration of drugs.

### Statistical analysis

Continuous variables were expressed as mean with standard deviation (SD) and categorical variables were expressed as percentages. Means and SDs were compared using Student´s t-test and non-parametric tests (Wilcoxon rank sum test).

Univariate analysis using Pearson chi^2^-test was used to test the association between DR and clinical parameters and macro- and microvascular disease (cf. Tables 1 and 2).

**Table 1:** Baseline characteristics of study participants.

	All patients (n = 1,255)			DR (n = 894)		Non-DR (n = 361)		*P*
Female	n (%)	578	(46.1)	421	(47.1)	157	(43.5)	0.247^2^
Age (years)	mean (± SD)	65.7	(8.3)	65.1	(8.0)	67.2	(8.7)	** < 0.001**^1^
≤ 50	n (%)	59	(4.7)	42	(4.7)	17	(4.7)	
51–64	n (%)	476	(37.9)	369	(41.3)	107	(29.6)	
65–74	n (%)	531	(42.3)	374	(41.8)	157	(43.5)	
≥ 75	n (%)	189	(15.1)	109	(12.2)	80	(22.2)	
Body mass index (kg/m^2^)	mean (± SD)	27.5	(4.8)	27.7	(4.8)	27.1	(4.8)	**0.040**^1^
< 25	n (%)	398	(31.8)	278	(31.1)	120	(33.2)	
> 25	n (%)	503	(40.2)	355	(39.7)	148	(41.0)	
> 30	n (%)	253	(20.2)	186	(20.8)	70	(19.4)	
> 35	n (%)	98	(7.8)	75	(8.4)	23	(6.4)	
Smoking status:								0.068^2^
Smoker	n (%)	108	(8.6)	69	(7.7)	39	(10.8)	
Non-smoker	n (%)	748	(59.6)	549	(61.4)	199	(55.1)	
Ex-smoker	n (%)	399	(31.8)	276	(30.9)	123	(34.1)	
Time on dialysis (months)	mean (± SD)	8.3	(5.9)	8.8	(7.2)	6.9	(5.9)	** < 0.001**^1^
**Macrovascular disease**								
Previous MI	n (%)	221	(17.6)	148	(16.6)	73	(20.2)	0.123^2^
Previous PTCA	n (%)	79	(6.3)	50	(5.6)	29	(8.0)	0.107^2^
Previous CABG	n (%)	100	(8.0)	69	(7.7)	31	(8.6)	0.607^2^
Overall-CAD (MI or PTCA or CABG)	n (%)	290	(23.1)	189	(21.1)	101	(28.0)	**0.009**^2^
Stroke / TIA	n (%)	224	(17.8)	161	(18.0)	63	(17.5)	0.815^2^
CHD	n (%)	265	(21.1)	190	(21.3)	75	(20.8)	0.851^2^
**Microvascular disease**								
PVD	n (%)	560	(44.6)	431	(48.2)	129	(35.7)	** < 0.001**^2^
PN	n (%)	753	(60.0)	630	(70.5)	123	(34.1)	** < 0.001**^2^
DG	n (%)	179	(14.3)	152	(17.0)	27	(7.5)	** < 0.001**^2^
NS	n (%)	392	(31.2)	318	(35.6)	74	(20.5)	** < 0.001**^2^

^1^Student’s t-test.

^2^chi^2^–test.

DR: diabetic retinopathy; MI: myocardial infarction; PTCA: percutaneous transluminal coronary angioplasty; CABG: coronary artery bypass graft; CAD: coronary artery disease; TIA: transitoric ischemic attacks; CHD: coronary heart disease; PVD: peripheral vascular disease; PN: polyneuropathy; DG: diabetic gangrene; NS: nephrotic syndrome.

**Table 2:** Baseline characteristics of study participants: blood pressure, glucose and lipid metabolism; hematology.

	All patients (n = 1,255)			DR (n = 894)		Non-DR (n = 361)		*P*
**Blood pressure**								
Hypertension	n (%)	1,114	(88.8)	807	(90.3)	307	(85.0)	**0.008**^2^
Hypertension stages:								
Normal	n (%)	97	(7.7)	62	(6.9)	35	(9.7)	
Pre-hypertension	n (%)	329	(26.2)		(25.7)	99	(27.4)	
Stage 1	n (%)	450	(35.9)	312	(34.9)	138	(38.2)	
Stage 2	n (%)	379	(30.2)	290	(32.4)	89	(24.7)	**0.032**^2^
Systolic BP (mmHg)	Mean (± SD)	145.6	(22.02)	146,7	(22.36)	142.7	(20.92)	**0.004**^1^
Diastolic BP (mmHg)	Mean (± SD)	75.8	(10.99)	76,2	(10.86)	75	(11.3)	0.077^1^
BP amplitude (mmHg)	Mean (± SD)	69.7	(18.74)	70,5	(19.12)	67.8	(17.63)	**0.017**^1^
**Glucose metabolism**								
T2DM duration								
prior to study (years)	Mean (± SD)	12.3	(1.92)	12,5	(1.84)	12.1	(2.06)	** < 0.001**^1^
Glucose (mg/dl)	Mean (± SD)	151.6	(5.86)	155,2	(53.34)	142.6	(42.88)	** < 0.001**^1^
HbA1c (%)	Mean (± SD)	6.7	(0.04)	6,9	(1.26)	6.3	(1.17)	** < 0.001**^1^
**Lipid metabolism**								
Cholesterol (mg/dl)	Mean (± SD)	219.3	(42.62)	219.6	(43.32)	218.4	(40.88)	0.639^1^
Triglycerides (mg/dl)	Mean (± SD)	263.9	(166.82)	259.4	(164.94)	275.1	(171.11)	0.133^1^
VLDL cholesterol (mg/dl)	Mean (± SD)	57.7	(33.88)	57.3	(34.09)	58.7	(33.37)	0.495^1^
LDL cholesterol (mg/dl)	Mean (± SD)	125.6	(29.86)	125.6	(29.84)	125.4	(29.96)	0.908^1^
HDL cholesterol (mg/dl)	Mean (± SD)	36.2	(13.19)	36.9	(13.74)	34.4	(11.56)	**0.003**^1^
**Apolipoproteins**								
Apo A I (mg/dl)	Mean (± SD)	126.3	(23.6)	127.4	(24.62)	123.7	(20.95)	**0.013**^1^
Apo A II (mg/dl)	Mean (± SD)	28.2	(5.8)	28.5	(5.96)	27.7	(5.22)	**0.050**^1^
Apo B (mg/dl)	Mean (± SD)	109.9	(29.7)	109.0	(29.38)	112.0	(30.38)	0.111^1^
Apo C II (mg/dl)	Mean (± SD)	6.3	(3.0)	6.4	(3.07)	6.3	(2.95)	0.601^1^
Apo C III (mg/dl)	Mean (± SD)	20.4	(9.5)	20.3	(9.53)	20.8	(9.44)	0.344^1^
Apo E (mg/dl)	Mean (± SD)	11.8	(4.0)	11.8	(4.07)	11.9	(3.91)	0.555^1^
**Hematology**								
Hb (g/dl)	Mean (± SD)	0.2	(0.4)	10.9	(1.34)	10.8	(0.72)	0.105^1^
Leucocytes (× 1,000/μl)	Mean (± SD)	8.1	(2.4)	8.0	(2.36)	8.3	(2.58)	**0.033**^1^
Platelets (× 1,000/μl)	Mean (± SD)	257.0	(80.6)	258.2	(81.06)	254.2	(79.75)	0.638^1^

^1^Student’s t-test.

^2^chi^2^–test.

DR: diabetic retinopathy, BP: blood pressure; T2DM: type 2 diabetes mellitus; VLDL: very-low-density lipoprotein; LDL: low-density lipoprotein; HDL: high-density lipoprotein; apo: Apolipoprotein; Hb: haemoglobin.

Multilevel mixed logistic regression models were used to assess associations of clinical and laboratory variables with DR. The models were adjusted for the basic clinical parameters (gender, age, BMI, smoking status and time on dialysis), macrovascular diseases (MI, PTCA, CABG, CAD, stroke/TIA and coronary heart disease (CHD)), microvascular diseases (PVD, PN, DG and NS), blood pressure (hypertension, systolic and diastolic blood pressure (BP) and BP amplitude), glucose metabolism (duration of T2DM, glucose and HbA1c), lipid metabolism (cholesterol, triglyceride (TG), very-low-density lipoprotein (VLDL), LDL and HDL cholesterol and apolipoproteins) and hematology (hemoglobin (Hb), leucocytes and platelets) (cf. Tables 3 and 4).

**Table 3:** OR and CI for retinopathy according to baseline characteristics of study participants.

	Univariate			Multivariate		
	OR	95% CI	*P*	OR	95% CI	*P*
**Clinical**						
Sex	1.25	0.96–1.63	0.098	1.34	0.99–1.84	0.063
Age (per year)	0.97	0.95–0.98	** < 0.001**	0.97	0.95–0.99	**0.001**
Age (per stratum)	0.70	0.59–0.83	** < 0.001**	0.70	0.59–0.83	** < 0.001**
BMI (per kg/m^2^)	1.03	1.00–1.06	**0.044**	1.01	0.98–1.05	0.477
Smoking	0.69	0.46–1.04	0.079	0.56	0.35–0.90	**0.018**
Time on dialysis (per month)	1.04	1.02–1.07	**0.001**	1.04	1.01–1.06	**0.004**
**Macrovascular disease**						
MI	0.79	0.56–1.10	0.156	1.29	0.62–2.70	0.498
PTCA	0.68	0.41–1.31	0.137	0.95	0.47–1.94	0.895
CABG	0.93	0.58–1.49	0.771	2.05	1.06–3.96	**0.034**
Overall-CAD (MI or PTCA or CABG)	0.68	0.51–0.92	**0.013**	0.69	0.50–0.96	**0.026**
Stroke / TIA	1.06	0.75–1.50	0.737	1.02	0.70–1.49	0.934
**Microvascular disease**						
PVD	1.71	1.30–2.24	** < 0.001**	1.24	0.89–1.71	0.203
PN	4.90	3.68–6.52	** < 0.001**	3.61	2.66–4.89	** < 0.001**
DG	2.63	1.68–4.12	** < 0.001**	1.46	0.89–2.41	0.139
NS	2.03	1.48–2.79	** < 0.001**	1.60	1.13–2.25	**0.008**

OR: odds ratio; CI: confidence interval, BMI: body mass index; MI: myocardial infarction; PTCA: percutaneous transluminal coronary angioplasty; CABG: coronary artery bypass graft; CAD: coronary artery disease; TIA: transitoric ischemic attack; CHD: coronary heart disease; PVD: peripheral vascular disease; PN: polyneuropathy; DG: diabetic gangrene; NS: nephrotic syndrome. The P-data which is shown bolded is statistically significant.

**Table 4:** OR and CI for retinopathy according to cardiovascular risk factors: blood pressure, glucose and lipid metabolism; hematology.

	Univariate			Multivariate		
	OR	95% CI	*P*	OR	95% CI	*P*
**Blood pressure**						
Hypertension	1.63	1.13–2.35	**0.008**	1.57	1.06–2.33	**0.024**
Hypertension stages						
Normal	0.61	0.43–0.88	**0.008**	1.05	0.77–1.42	0.757
Pre-hypertension	0.92	0.70–1.21	0.536	0.76	0.58–1.00	0.052
Stage 1	0.87	0.67–1.12	0.266	1.53	1.13–2.07	**0.006**
Stage 2 (full model)	1.64	1.11–2.44	**0.014**	1.51	0.98–2.35	0.063
Systolic BP (per mmHg)	1.01	1.00–1.01	**0.013**	1.01	0.99–1.02	0.240
Diastolic BP (per mmHg)	1.01	0.99–1.02	0.086	1.01	0.97–1.02	0.154
BP amplitude (mmHg)	1.01	1.00–1.01	0.058	0.997	0.981–1.013	0.981
**Glucose metabolism**						
T2DM duration (per year)	1.08	1.060–1.099	** < 0.001**	1.064	1.045–1.085	** < 0.001**
Glucose (per mg/dl)	1.01	1.003–1.008	** < 0.001**	1.006	1.002–1.009	**0.001**
HbA1c (per %)	1.53	1.356–1.723	** < 0.001**	1.613	1.405–1.852	** < 0.001**
**Lipid metabolism**						
Cholesterol (per mg/dl)	1.00	0.99–1.00	0.664	1.00	0.97–1.00	0.922
Triglycerides (per mg/dl)	1.00	0.99–1.00	0.248	1.00	0.99–1.00	0.065
VLDL cholesterol (per mg/dl)	0.99	0.99–1.00	0.488	1.00	0.99–1.00	0.804
LDL cholesterol (per mg/dl)	1.00	0.99–1.00	0.976	0.99	0.98–1.00	0.111
HDL cholesterol (per mg/dl)	1.02	1.00–1.03	**0.002**	1.02	1.01–1.03	**0.004**
**Apolipoproteins**						
Apo AI (per mg/dl)	1.01	1.00–1.01	**0.010**	1.00	0.99–1.01	0.326
Apo AII (per mg/dl)	1.02	0.99–1.05	0.068	1.01	0.98–1.04	0.387
Apo B (per mg/dl)	0.99	0.99–1.00	0.106	0.99	0.98–0.99	**0.012**
Apo CII (per mg/dl)	1.01	0.97–1.06	0.540	1.05	0.97–1.13	0.248
Apo CIII (per mg/dl)	0.99	0.98–1.01	0.426	0.99	0.96–1.01	0.269
Apo E (per mg/dl)	0.99	0.96–1.03	0.799	1.01	0.96–1.06	0.655
**Hematology**						
Hb (per g/dl)	0.94	0.890–0.990	**0.020**	1.08	0.98–1.19	0.129
Leucocytes (× 1,000/μl)	0.95	0.90–0.99	**0.033**	0.93	0.87–0.99	**0.019**
Platelets (× 1,000/μl)	1.00	0.998–1.003	0.754	1.00	0.99–1.00	0.754
CRP (mg/l)	0.99	0.98–0.99	**0.001**	0.98	0.97–1.00	0.052

OR: odds ratio; CI: confidence interval, BP: blood pressure; T2DM: type 2 diabetes mellitus; VLDL: very-low-density lipoprotein; LDL: low-density lipoprotein; HDL: high-density lipoprotein; apo: Apolipoprotein; Hb: hemoglobin; CRP: C-reactive protein. The P-data which is shown bolded is statistically significant.

Full model includes all predictors, while the final model includes only predictors that emerged significantly associated with DR in the full model. Only results of the unadjusted and final adjusted model are reported. All p-values are reported two-sided. Analysis was performed using STATA (StataCorp LLC, College Station, TX, USA).

Type I error inflation by multiple testing is a common issue in epidemiological studies. We have implemented the following type I error, controlling measures in the present analysis: First, for each potential categorical risk factor with more than two categories, e.g. the four age strata (≤ 50; 51–64; 65–74; ≥ 75 years), we considered the overall Wald statistic simultaneously testing the global hypothesis whether any of the contrast parameters was significantly different from zero. Secondly, in testing the risk association of each potential risk predictor we faced a ‘multiple hypotheses scenario’ rather than a ‘multiple testing scenario’. We considered each test of a multivariate adjusted risk association as a different hypothesis test for a different risk process. The impact of other covariates is addressed by simultaneously including all factors as predictors in the models and thus also decreasing the degrees of freedom of the respective hypothesis test.

### Ethical approval

All procedures performed in this study were in accordance with the ethical standards of the institutional committee, with the 1964 Helsinki declaration and its later amendments or comparable ethical standards.

### Informed consent

Informed consent was contained from all individual participants included in this study.

## Results

During March 1998—October 2002, 4D study included a total of 1,255 patients with T2DM related hemodialysis. The current results represent a cross-sectional analysis of baseline characteristics of the study participants.

For the complete group of participants, the results of the baseline characteristics, which are shown in Tables 1 and 2, were as follows:

The mean age of the study population was 65.7 years, 54% of the patients were male. Most patients had a BMI in the range of 20 to 30 kg/m^2^; the mean BMI of all patients was 27.5 kg/m^2^. About 9% of patients were current smokers, 32% were former smokers, and 59% non-smokers. The mean time on dialysis was 8.3 months. Nearly 89% of all patients suffered from hypertension (including pre-hypertension). The mean systolic BP was 145.6 mmHg, mean diastolic BP 75.8 mmHg and mean BP amplitude 69.7 mmHg. The overall mean duration of T2DM was 12.3 years, mean glucose was 151.6 mg/dl and mean HbA1c was 6.7%. DR was present in 71% of the patients. Blindness, defined as a visual acuity lower than 3/60^7^, was found in 6.1% (83 right and 71 left eyes).

We could not find a gender difference between the DR and non-DR group. BMI was only slightly higher among patients with DR compared to those without and there was no statistical difference in smoking habits (current smoker, former smoker, non-smoker).

Considering, that all statistical test-methods (t-test/chi^2^-test, uni- and multi-variate OR; cf. Tables 1–4) showed statistically significant results, we found the following differences between DR and non-DR group: There was a highly negative correlation between DR and age: older patients were more common in the non-DR group (cf. Tables 1 and 3; multivariate OR 0.97). The time on dialysis was significantly longer in patients with DR compared to those without (cf. Tables 1 and 3; multivariate OR 1.04). In regards to macrovasculopathies, in the DR group only overall-CAD (MI or PTCA or CABG) was less prevalent than in patients without DR (cf. Table 3; multivariate OR 0.69). There was no statistical difference in the prevalence rates of previous MI, PTCA, CABG and stroke/TIA between patients with DR and without. In regards to microvasculopathies, the prevalence rates of PN and NS were greater in patients with DR compared to those without (cf. Tables 1 and 3; multivariate OR 3.61 and 1.60).

The prevalence of hypertension was overall greater among patients with DR compared to those without (cf. Tables 2 and 4; multivariate OR 1.57). Furthermore, the stage 2 of hypertension was more frequent in the DR group (t-test, univariate OR) and the systolic BP was higher in the DR compared to the non-DR group (t-test, univariate OR). Mean diastolic BP was not different between the DR and non-DR group, however, the mean BP amplitude was higher in DR (t-test).

The duration of T2DM was longer, the levels of glucose and HbA1c were higher among patients with DR compared to those without (cf. Tables 2 and 4; multivariate OR 1.06, 1.01 and 1.61).

The mean concentration of HDL cholesterol in the DR group was higher than in the non-DR group (cf. Tables 2 and 4; multivariate OR 1.02). However, we could not find any association of cholesterol, TG, VLDL cholesterol or LDL cholesterol with DR (cf. Tables 2 and 4). There was a higher level of apo AI in the DR group (t-test, univariate OR). Mean apo AII was also slightly higher in DR (t-test). The levels of apo B, CII, CIII, and E were not significantly different between (cf. Tables 2 and 4) the DR and non-DR group.

The mean leucocyte count was higher in the non-DR patients compared to DR (cf. Table 2). Consistently, multivariate OR showed a slightly negative association of leucocytes and C-reactive protein (CRP), although marginal not statistically significant (cf. Table 4; OR 0.93 and 0.98). No difference were found for Hb and platelets (cf. Tables 2 and 4).

## Discussion

This is the largest study so far, addressing DR in patients with T2DM on hemodialysis. We were interested to investigate the prevalence rate of DR in T2DM hemodialysis patients, in clinical and biochemical differences between patients with and without DR.

### Prevalence rates of DR

The prevalence of any stage of DR was 71% in our patients. Even compared to other studies of DR in hemodialysis patients, our prevalence of DR is higher: El-Menyar et al.^12^ found 113/252 (45%) DR in hemodialysis patients and Vrabec et al.^13^ reported 5/64 (7.8%) DR in hemodialysis patients. Lee et al.^16^ reported 21.6% proliferative DR (PDR) and 13.7% high-risk PDR in patients with DR and chronic kidney disease (CKD). The prevalence rate of DR in T2DM-patients not receiving dialysis is reported at 34.6% by Olafsdottir et al.^5^ or 64% by Tomic et al.^6^ or, respectively. Sasongko et al.^17^ reported in 224 patients with type 1 or type 2 DM, not receiving dialysis, DR in 64% of patients and vision-threatening DR in 25%. This shows that prevalence rates vary over wide range. However, we consider the rate of 71% reasonable, because it is slightly above the highest figures reported for DM-patients not receiving dialysis^17,18^. It may also be related to thorough clinical characterization of the 4D patients on inclusion to the study. The high frequency rate of DR in hemodialysis patients points out a common pathophysiological denominator of changes in retinal and renal vessels^11,19^.

### DR and blindness

DR is known as the most prevalent cause for blindness in working-age people in developed countries^7^. *Blindness* is defined as visual acuity lower than 3/60, as described above, and *severe visual impairment* is defined as a visual acuity lower than 6/18 but greater than 3/60^7^. Using these definitions in the general population worldwide, the rate of visual impairment and blindness is 1% due to DR^7^. Flaxman et al.^20^ described, in adults aged ≥ 50 years in central Europe, a prevalence rate of moderate/severe visual impairment or blindness due to DR of 3.12% (0.32–7.43) or 3.1% (0.27–7.33), respectively. In our patients, 6% of the eyes observed blindness, which is nearly double of the rate reported by Flaxman et al. The rate of high-risk PDR reported by Lee et al.^16^ was greater than 13%. Considering, that approximately half of the patients with a high-risk PDR get severe visual impairment^21^, the results of Lee seem to be consistent with our findings. Overall, comparison of these research studies with ours is difficult, because there is no data on the proportion of DR stages in dialysis patients available.

### Smoking

We found no association of smoking habits (current smoker, former smoker, non-smoker) with DR, which could be due to unadmitted smoking. However, also under-reporting of smoking occurs in clinical studies^22^, it has been found to be in the range of 3.4%. Therefore, we do not believe that it caused a major bias in the current study.

It is well known that cigarette smoking is a risk factor for atherosclerotic diseases and the progression of DM^23^. The correlation between cigarette smoking and DR is unclear. Some researchers found no association between smoking and DR^24^ and in one study even a “protective” effect was seen^25^.

### Common findings for the DR-group

DR was associated with hypertension, BMI, poor glucometabolic control, the duration of T2DM, dialysis and the prevalence rates of other microangiopathies (PN, DG, and NS).

In line with other publications, we found a positive correlation between DR and hypertension: The UKPD study demonstrated, that patients with T2DM received a considerable benefit from BP lowering^25^. High blood pressure is a major risk factor for atherosclerosis in general, in T2DM and in DR^26^. Studies showed that blood pressure lowering also leads to reduced progression of DR^27^. As our data is consistent with the available research. To emphasize, however, that we merely report an association study and that a blood pressure lowering intervention has not been performed.

The significant correlations between DR, glucose, DM duration, and most notably HbA1c^27^ were not unexpected. It is well established that glucose and HbA1c are linked to the progression of DR and that lowering glucose and HbA1c may slow down the development of DR^28,29^. However, rigorous glucose lowering may lead to episodes of hypoglycaemia and threaten the life of patients with diabetes mellitus^30^. Hypoglycaemia episodes trigger retinal proliferation. For patients suffering from DR, it is challenging to define an optimal HbA1c^31–33^.

We found a strong positive association between other kinds of microangiopathies (PVD, PN, DG, NS) and DR. This is consistent with previous evidence^34,35^, indicating that different microvasculopathies can share common risk factors.

### Unexpected findings for the DR-group

Surprisingly, patients without CAD rather than those with CAD, patients with high concentrations of HDL cholesterol, apo AI, apo AII, and, finally, those with lower systemic inflammation (as assessed by leukocyte counts) were more likely to have DR.

We had not expected, that patients without CAD were more prevalent to DR. Our findings are in contrast to Cheung et al.^36^, who described that DR was associated with a two times higher risk of incident CAD events and a three times higher risk of fatal CHD and Simó et al.^37^, who reported DR as an independent predictor of subclinical cardiovascular disease. Our finding may differ due to two reasons: Firstly, T2DM patients developing macrovascular disease might die before they develop significant DR or ESRD. Secondly, T2DM patients, who develop microvascular end organ damage, might differ from those developing macrovascular diseases with regard to genetic and metabolic factors. It is consistent with this concept that statin treatment, which mainly protects from macrovascular disease, has limited benefit in ESRD and causes of death in ESRD are different from those in post MI patients^15,38^.

### DR and inflammation

It is well known that local inflammation in the eye plays an important role in the development of DR. Multiple intravitreal factors were found to be elevated in patients having DR^39^. Systemic inflammatory factors are of interest, as well. For example, Sasongko et al.^17^ described that higher CRP levels may be related to more severe DR and that inflammatory processes are involved in severe DR, particularly in patients with a BMI ≥ 30 kg/m^2^. On a cellular level, leucocytes in DR patients show an increased retinal leukostasis, as shown by Joussen et al.^40^. Chibber et al.^41^ described that leucocytes in patients with DM are less deformable, more activated and have increased adhesion to vascular endothelium. Generally, high inflammatory level drives often increase or severe DR.

Here, in contrast, mean leucocyte counts were surprisingly higher in patients without DR compared to those with DR and there was a negative correlation of leucocytes and CRP with DR (cf. Tables 2, 4 and 8). These findings are in agreement with Lim et al.^42^ who found that higher levels of CRP are inversely related to DR.

One explanation is the apparent paradox that leucocytes and CRP are markers, reflecting systemic inflammatory burden, rather than the inflammatory activity within the eye. It is more likely that subclinical inflammation is contributing to DR rather than DR to be the cause of subclinical inflammation. Data to substantiate this assumption has not been collected because this was an epidemiological rather than an experimental study.

### DR and lipids

Surprisingly, we also found higher concentrations of HDL cholesterol and the major HDL apolipoproteins (apo AI and AII) in DR patients, while other measures of lipid metabolism showed no correlation.

It is well known that high concentrations of apo B containing lipoproteins (VLDL, remnants, LDL) increase the risk for atherosclerotic vascular disease in diabetes mellitus^43,44^. Their link with DR remains unclear: some^29,45^, but not all authors^46^, have found DR related to higher TGs and that lowering of TG by fenofibrate may have a positive effect on DR in patients not receiving dialysis.

Previous research on the correlation of HDL metabolism with DR is also heterogeneous. Studies show, HDL cholesterol was not^46,47^ or not significantly^48^ correlated to DR. On the other hand, Toth et al.^49^ showed that high HDL cholesterol reduces the risk of microvascular complications and Sasongko et al.^50^ found that lower levels of HDL cholesterol and apo AI are associated with DR. Consistently, Hu et al.^51^ showed that a lower apo AI levels and a lower apo AI to apo B ratio were significantly associated with the more severe type of proliferative DR. Reviewing article, Chang et al.^52^ reported 13 studies dealing with DR and lipids. In 3 studies no data on HDL were available, 8 showed no association between HDL and DR, and two studies reported an association with HDL and DR: In UKPDS 30^53^ a positive correlation of HDL-concentrations and the severity of DR was found whereas Popescu et al.^54^ described a negative correlation of HDL and the appearance of DR.

It is becoming clearer that the anti-atherogenic properties of HDL are not completely reflected by the concentration of HDL cholesterol. For instance, increasing HDL cholesterol by inhibition of cholesteryl ester transfer protein has so far not reduced cardiovascular endpoints^55^. The same is true for increases in HDL cholesterol induced by nicotinic acid^56,57^.

Therefore, the seemingly paradox association of high HDL cholesterol with DR may indicate, that in dialysis patients dysfunctional HDL particles are accumulating which lack the well-known beneficial effects of HDL (stimulation of endothelial NO, promotion of macrophage cholesterol efflux, anti-inflammatory and anti-thrombotic effects)^58,59^.

In summary, we found that more than two-thirds of patients with T2DM receiving hemodialysis suffered from DR. As expected, patients with DR had statistically longer time on dialysis and duration of diabetes. Glucometabolic control, hypertension, higher BMI, lower leucocytes concentrations and other microvasculopathies were associated with DR. Surprisingly, patients without CAD were more likely to have DR. Paradoxically, high concentrations of HDL (HDL cholesterol, apo AI, and apo AII) were seen in DR. While macrovascular disease in DM is primarily driven by disorders of lipid metabolism, microvascular disease is likely connected to glycemic control. This was illustrated by the UKPDS 35 study^60^: per 1% increase in HbA1c the risk of microvascular complications increased by 37%, while the risk of myocardial infarction was only increased by 14%. Our results stand in line with this concept.

When we look at the clinical utility of our above-mentioned findings, we have to consider that a difference of 2 months longer in time on dialysis and 3.3 months longer for T2DM duration between the DR and non-DR group is irrelevant in clinical utility. In addition, same might be true for the difference regarding the leucocyte counts and HDL concentrations between the two groups. All of the above-mentioned parameters are not (time on dialysis or T2DM duration) or difficult to affect. Surprisingly, even in the DR group the HbA1c level was lower than 7%, which is the clinical aim to avoid DR or DR-progression^28,29^. In the non-DR group the mean HbA1c was 6.3%, which is clinically hard to achieve (holds the hazard to hypoglycemia and cardiac side effect). Although, due to our study design, we are not able to deflect therapeutic recommendations, we would warrant careful control of blood pressure, glucose metabolism and weight reduction in order to potentially prevent DR progression in T2DM patients undergoing hemodialysis. These are the only alterable parameters we found more pronounced in the DR-group.

### Study limitations

This study has limitations, it was a post-hoc analysis within a selected cohort of German patients with T2DM on hemodialysis. Therefore, our results may not apply to other patient populations. Heterogeneity of patient characteristics across the recruiting centers cannot completely be ruled out, as the study was conducted in Germany only. Given that 178 centers were involved and that many centers contributed only a few patients, we could not statistically identify meaningful differences between the centers. In addition to our statements regarding statistical data evaluation in the “material and methods” section we suggest, that multiple testing for associations inflates type I error, what might limit the power of our findings.

The diagnosis of DR was not based on our own ophthalmological examinations and there was no differentiation of the DR stages and severity. We are not able to distinguish between (mild, moderate or severe) non-proliferative or proliferative DR. Thus, we are not able to explore how the risk factors may have aggravated the severity of DR, which is without question a limitation of our study. We attempted to focus on metabolic risk factors of DR. Therefore, we did not analyse dialysis-related factors like increase of body weight during the dialysis, episodes of hypotension, or dialysis adequacy. Medication use in the DR and the non-DR groups was not adjusted for. Another limitation of this study, is due to cross-sectional rather than prospective by design. Therefore, causalities and longitudinal temporal relationships cannot be proven. Furthermore, we are not able to delineate therapeutic recommendations from the data, because our study was observational rather than interventional.

## Data Availability

Due the consent given by the study participants, data cannot be released to the public domain. Data shall be made available to researchers upon request and approval by the principal investigator Christoph Wanner MD. Any exploitation of the data needs to make sure that rules of good scientific practice are followed and that credit is given to the people who have been in charge of the design and the organization of the study. Interested researchers are invited to address their request or proposal to Christoph Wanner (Wanner_C@ukw.de). The authors confirm that they accessed these data upon approval by Christoph Wanner and that all other researchers can access the data in the same manner the authors did.
